# Design, Implementation and Evaluation of an Indoor Navigation System for Visually Impaired People

**DOI:** 10.3390/s151229912

**Published:** 2015-12-21

**Authors:** Alejandro Santos Martinez-Sala, Fernando Losilla, Juan Carlos Sánchez-Aarnoutse, Joan García-Haro

**Affiliations:** Department of Information and Communication Technologies, Universidad Politécnica de Cartagena, Campus Muralla del Mar, Cartagena E-30202, Spain; fernando.losilla@upct.es (F.L.); juanc.sanchez@upct.es (J.C.S.-A.); joang.haro@upct.es (J.G.-H.)

**Keywords:** UWB, Ubisense, indoor navigation, visually impaired, SUGAR

## Abstract

Indoor navigation is a challenging task for visually impaired people. Although there are guidance systems available for such purposes, they have some drawbacks that hamper their direct application in real-life situations. These systems are either too complex, inaccurate, or require very special conditions (*i.e.*, rare in everyday life) to operate. In this regard, Ultra-Wideband (UWB) technology has been shown to be effective for indoor positioning, providing a high level of accuracy and low installation complexity. This paper presents SUGAR, an indoor navigation system for visually impaired people which uses UWB for positioning, a spatial database of the environment for pathfinding through the application of the A* algorithm, and a guidance module. The interaction with the user takes place using acoustic signals and voice commands played through headphones. The suitability of the system for indoor navigation has been verified by means of a functional and usable prototype through a field test with a blind person. In addition, other tests have been conducted in order to show the accuracy of different relevant parts of the system.

## 1. Introduction

According to the latest data released by the World Health Organization (WHO) [1], over 285 million people are estimated to be visually impaired worldwide. One of the main challenges that this demographic has to face in their everyday lives is orientation and mobility in both outdoor and indoor scenarios. Outdoor navigation is adequately dealt with by the Global Positioning System (GPS). Unfortunately, GPS receivers cannot be used for indoor navigation because satellite signals do not easily penetrate inside buildings and, therefore, indoor navigation continues to be a challenging issue.

Several projects have addressed the problem of indoor navigation for visually impaired people in many different ways [2,3,4,5,6,7,8]. However, as will be seen, they have some drawbacks that jeopardize their practical usability. Current indoor navigation systems tend to require either complex installations or very specific conditions that are hard to encounter in real life. The positioning technologies on which they rely, respectively, either use short-range signals, and therefore require deploying a considerable number of devices, or are heavily affected by the lack of direct line of sight between devices, multipath interference, or error accumulation among other issues that are detrimental to accuracy, which is essential for the guidance of people with severe visual impairment. In this regard, the use of a long-range high-precision positioning technology able to operate in the presence of obstacles and multipath signal reflections from walls, furniture or people would greatly improve the performance of the system.

Among the many different technologies and techniques which may be used for positioning, Ultra-Wideband (UWB) stands out for the high accuracy it can achieve in indoor environments [9]. UWB uses narrower radiofrequency pulses than other RF technologies, which facilitates distinguishing direct path signal carrying useful information for positioning from reflections. In addition, the presence of obstacles may have a major impact on the Received Signal Strength Indicator (RSSI); however, UWB in combination with methods estimating position according to the time or angle of arrival will not be so affected. Therefore, UWB-based positioning technologies can be quite robust and, having a transmission range greater than other competing technologies such as RFID, enable less complex deployments with fewer devices. In this study, the Ubisense [10] UWB-based commercial solution has been used to obtain periodic location updates, which, combined with other orientation information acquired by a smartphone, are processed in order to improve accuracy, and to serve as input for other system modules in charge of planning routes and guiding the user.

The system developed, called Sistema Universal de Guiado Avanzado en Recintos cerrados (SUGAR, Universal Advanced Guidance System in Enclosed Areas), is aimed not only at providing high accuracy in terms of location, but also a relatively simple deployment and ease of use for visually impaired people. From the user’s perspective, only a few simple and light devices are required, such as small UWB tags and other everyday objects such as smartphones and headphones, which enable a natural interaction with the user through different acoustic signals and voice commands. From the administrator’s perspective, the system also requires the installation of a few sensors that are used to estimate the location of the users and to follow several simple calibration and initialization steps. In addition, given the range of the positioning system, the system can be used in large areas consisting of one or more rooms. A user of the system may find a room as well as points of interest within the room. This might be of special interest in public buildings and venues that host conferences, meetings, or a range of events.

The rest of the paper is organized as follows: Section 2 introduces related work in the field of indoor navigation and outlines the main disadvantages that make them unsuitable for the scenario proposed in this paper. Section 3 describes our current proposal, focusing on the system architecture. Section 4 details and discusses the results of a field test and additional accuracy tests that were performed. Section 5 concludes the work.

## 2. Related Work

Over the last few years, different approaches have been proposed in order to provide a reliable and accurate indoor positioning, guidance, and navigation system. Some of these proposals were conceived specifically for blind or visually impaired people. Giudice *et al*. [11] surveyed several Electronic Travel Aids (ETAs) for blind people. In the next paragraphs, we will briefly overview the main aspects considered in the related literature regarding the different indoor positioning systems aimed at blind or visually impaired people that have emerged in the last few years.

Tsirpas *et al*. [2] proposed an interesting indoor navigation system for visually impaired and elderly people based on Radio Frequency Identification (RFID). The main weakness of RFID-based systems is that RFID tags only provide a short range (in their study, the authors suggest cells of 40 × 40 cm). Moreover, this range could be shorter if we consider that the human body can block RF signals [12]. Another disadvantage is that its implementation in large environments may be costly, since, in most cases, RFID tags need to be embedded into objects, furniture, floors, or even walls.

A similar approach has been developed with digitally-encoded signs [13]. In this work, the user has to handle a sign-reader to read the signs that are widely distributed throughout a building. This system is not suitable for our scenario, because: (1) in certain situations, there can be several signs close enough to confuse the user; (2) the system requires a direct line of sight that cannot always be accomplished (other people, objects, *etc*. can be blocking the reader); and (3) the accuracy obtained in the experiments carried out in this work depends substantially on the user (how much they can see, their age, *etc*.). Furthermore, the signs can be read up to a maximum distance of approximately 2.5 m, and within an angle of 60°, and with this information the location estimation cannot be as accurate as our scenario requires. In addition, the authors admit that finding and reading the tags non-visually is more challenging in an open space, because the location of the tags can be less predictable.

Inertial dead reckoning navigation systems make use of inertial sensors to calculate the user’s position when a known starting point and a measured gait length are given. However, these systems suffer from error accumulation, which makes them an invalid solution for our scenario. Trying to solve this problem, Riehle *et al*. [14] proposed to use the trajectory of a planned route to provide additional information to reduce the error accumulation problem. The main drawback of this idea is that the user has to follow a fixed route, and in our scenario (museums, conferences, *etc*.) the user should be free to move around the facilities.

A more complex system was conceived by Hub *et al*. [15]. These authors proposed adding RFID technology to their original navigation prototype called Tactile-Acoustical Navigation and Information Assistant (TANIA). This first prototype was developed to try to deal with both indoor and outdoor navigation by means of GPS, movement sensors, and detailed maps of the environment. In the enhanced version, an RFID reader is added in order to determine the initial position of the user when the GPS position is not available. Obviously, the position given by the RFID reader can also be used to retrieve the position of the user at a certain point (this way, the problem of the inertial sensor can be reduced). Despite the appropriateness of this system, it is not suitable for our scenario, because: (1) GPS is not available in indoor situations; (2) inertial sensors suffer from error accumulation that cannot be completely solved if the user does not reach an RFID tag; and (3) the RFID reader’s range is quite short and, therefore, the user has to get close enough to the tag (but within an error radius, meaning that it is not that accurate) or there have to be enough tags to warrant good coverage (again, this may prove costly).

In [3], Guerrero *et al*. presented a micro-navigation system [4] designed to provide navigation support by means of detecting the user’s position and movement intentions in addition to the presence of all the objects and/or obstacles in the surrounding area. In order to do so, the system captures the user’s position and movement through infrared cameras.

In this system, the user must carry an augmented white cane with several embedded infrared lights. Therefore, the user's location and movement are tracked when more than one IR camera are able to capture the IR LEDs of the cane. This tracking information is used by a software application running on a computer to determine the user’s position and movement. To avoid obstacles, the software application retrieves all the parameters required to describe the room in question and the objects contained on it (the room’s dimensions, positions of obstacles, positions of cameras, *etc*.) from an XML file.

In our opinion, all these types of solutions present several drawbacks that make them unviable systems for the requirements of our application scenario. First, there must be a direct line of sight; if the cameras cannot detect the LEDs, the system does not work. Therefore, the cameras have to be placed in proper places to avoid non-visible areas. Furthermore, any kind of obstacle (objects in the room, people, or even the user) can block the line of sight. Another issue to consider in the use of IR is that daylight (and other light sources) can cause interference in IR cameras [16]. Another disadvantage of this system is the number of users it can support. The authors of this work recognized that the support for multiple users was not formally considered in this version of the system.

Nakajima and Haruyama [5] proposed the use of visible light communication technology and a smartphone that receives information transmitted by LEDs mounted in the ceiling of a room. Visible light communication is a communication technology that enables information to be sent by means of the lights of a building. This information can be, for instance, position information. The authors admitted that it is only possible to identify the user’s position within a range of 1–2 m, which is not accurate enough for our scenario.

Other authors, such as Cecilio *et al*. [6] developed systems based on smartphones and wireless sensors. These systems triangulate the position of the user by employing the signal strength of each wireless base station placed in the surroundings. Unfortunately, this technique shows low and unreliable precision due to multipath reflection. Furthermore, there should be a sufficient number of base stations to guarantee coverage and a training phase is also usually required where the smartphone has to measure the received signal strength at different locations in order to create a map.

Following a different approach, several authors have proposed robotic systems to assist or to guide visually impaired people [7,8]. Most of them consist of a robotic platform with different sensors (cameras, laser range finders, sonar, *etc*.) to detect obstacles and guide the user through facilities. Despite the use of complex navigation systems (Evolving Neural Controllers, *etc*.), these systems are less accurate than the solution proposed in our work. Additionally, they introduce a new element into a room, which makes it less scalable for use by several users in a shared area.

Although several drawbacks have been mentioned regarding the proposals described in this section, all of them present quite interesting systems that perform properly in the scenarios considered by the authors of the research. Consequently, these systems can be appropriate for small areas [2,3], low budget requirements [3,6], or environments with obstacles whose position may change [3,7,8]. However, the key requirements for our scenario are accuracy and reliability in large areas. In this regard, in [9,17], the authors compared the values achieved by different technologies. These two works establish that the most accurate technology is UWB, and among the different available UWB systems Ubisense offers the best performance.

In the previous paragraphs we have focused on the positioning techniques used because of their novelty or because they are representative of other similar research; however, pathfinding and guidance are other important parts of indoor navigation systems and there are also several remarkable approaches which are worth mentioning in this respect. Accordingly, the creation of a detailed spatial database from a building’s blueprints or other design documents is of special interest for visually impaired people, as it allows for the better detection of obstacles and the calculation of safer paths. In previously cited research, this is done in [2], where a grid structure of 40 × 40 cm squares is created by interpreting the blueprints of a building, in [3], where an XML file describes the layout of the environment, and in [6], where additional XML files describe services and products of interest that exist in the environment. 

In the same way, the aforementioned TANIA system [15] also makes use of detailed maps of the environment to provide additional information of the surrounding. The TANIA system map allows the administrator to store text information for landmarks than can be reproduced acoustically or in Braille.

In [18], Falco *et al*. also considered the use of detailed spatial databases but presented another possibility, with a system that is not only aimed at visually impaired people but at elderly people in general. Since they may have different disabilities, the authors proposed providing custom routes according to the special needs of each user.

There are also some projects which make use of Building Information Modeling (BIM) models, although none of them specifically focus on visually impaired people. BIM is a technology that is gaining momentum in construction which enables the design and management of buildings by means of models that not only take into account their geometry but also their functional characteristics. For instance, these models offer information such as where doors, stairs, and other elements are in a way that can be interpreted by a machine. Some proposals import this semantic data from the building’s design files, which allows the pathfinding process to be improved; for example, by taking into account whether doors are opened or closed [19] or, in the case of firefighters, by guiding them to fire detectors [20].

Finally, it is very important for the success of positioning systems that the needs and requirements of blind and visually impaired users are taken into account, as explained in [11]. Vision conveys much more information than any other sensory modality, which puts blind people at a disadvantage compared to sighted people. They must perform a myriad of tasks in order to navigate safely, which, unlike sighted people, requires the use of cognitive and attentional resources. Consequently, a challenge for any navigation system is reducing the cognitive load as much as possible. In addition, blind people, especially early blind and born-blind people, encode spatial information in a different way than sighted people. This has been studied by Thinus-Blanc *et al*. [21]. One of their conclusions is that this group of people require more cognitive effort to construct map-like representations, although it easier for them to encode sequential features of a travelled route (sequences of instructions that specify change of direction while traveling).

The above statements are very important in terms of the way that the system interacts with the user, since different technologies will entail different cognitive loads and effectiveness. Loomis *et al*. [22] performed several tests with a device that provides auditory or haptic information in order to compare the performance of different display technologies. In a first test, they compared speech with virtual sounds (the number of the next waypoint was perceived from the direction of the waypoint). The results showed that virtual sounds had better performance than speech telling the user to turn left or right, but only slightly better performance than speech indicating how many degrees to turn in order to face the next waypoint. In a second test, they used a haptic pointer device to compare travel times for five different operation modes. They observed that modes with virtual sounds had better travel times due to less time taken in the reorientation phase once a waypoint was reached. In addition, they noticed that it is easier for visually impaired people to identify a direction relative to their bodies than to the orientation of the hands. Following a similar line, Klatzky *et al*. [23] evaluated the cognitive load of virtual sound in comparison with spatial language commands. For this purpose, they carried out several tests in which blindfolded users were asked to travel on a simulated pathway while simultaneously performing other cognitively demanding tasks. The results showed that the travel times and distances traversed associated with corrections given by language where higher than virtual sound due to the cognitive load associated with interpreting speech commands.

Finally, there are other devices that allow interaction with the user by means of haptic or acoustic feedback. Poppinga *et al*. [24] investigated whether it is possible to make a digital map on a touch screen device accessible to blind users by means of combining vibration feedback with speech that is triggered as the finger moves over relevant map objects. Their study concluded that interpreting a map in this way is a cognitively demanding task, as participants needed up to 15 min to make a copy of a map. Palani *et al*. [25] performed research along a similar line, tackling the need to perform non‑visual panning operations in order to access large format graphical information using vibration, audio, and kinesthetic cues. Experiments were performed on 15 blindfolded participants who learned a corridor layout map and performed subsequent spatial testing and reconstruction tasks. They concluded that using panning operations optimized for non-visual use caused no detrimental effects on the cognitive representation of the graphics tested, and that in some cases the use of panning improved the performance accuracy of the accomplished tasks. While these kinds of devices may be useful in order to learn new environments, their use in a guidance system poses some drawbacks, because, in addition to occupying one hand (while the other is supposed to hold the cane), receiving guidance information would require more attention from the user.

## 3. The SUGAR System

The proposed SUGAR system has been conceived and designed to provide accurate user positioning and guidance information for visually impaired people in indoor environments. As it will be reasoned in the following paragraphs, the system is specially targeted at large-scale deployments within large rooms. This may be the case of public buildings and venues that host conferences, meetings, or a range of events. In addition, in these scenarios, technical staff may be in charge of the installation and administration of the system.

Firstly, the way in which the SUGAR system overcomes the limitations of the other existing systems mentioned in the previous section will be explained. Accuracy is an important feature of the SUGAR system, which the selection of UWB technology for positioning improves to a great extent [9]. The accuracy level shown by the system is related in large part to the use of the UWB-based Ubisense commercial system, which claims a precision of up to 15 cm with a 95% confidence interval. The estimation of the error of our system, in turn, shows positioning errors up to 20 cm in most locations. The reasons for this small decrease in precision are explained in Section 4. In any case, the magnitude of the positioning error is low enough to enable the proper operation of the system, especially considering that the coordinates provided by Ubisense will be mapped to a grid of square nodes where the sides of each node will be bigger than the positioning error.

Other systems that rely on technologies such as RFID or NFC require the deployment of numerous devices in order to achieve a precision comparable to that obtained with SUGAR. Deploying devices every, for example, 80 cm, in each direction could lead to the use of hundreds of them in large deployments. In addition, their installation under the floor of rooms is a very expensive process, even more expensive than the devices themselves. Consequently, many systems using these technologies only place devices in strategic places such as the entrances to rooms. In SUGAR the cost of the devices is quite a bit higher than in these technologies, but very few of them are used, typically four sensors per room in addition to the devices attached to each user. Consequently, the SUGAR system is better suited for large-scale deployments, but may be more expensive in smaller ones. More specifically, it is a very good solution for buildings with large rooms, as the cost is dependent on the number of rooms but not on their size. In this regard, the range of the sensors is above 50 or 60 m (depending on the characteristics of the environment). Therefore, rooms whose side lengths are below 100 m can operate with a single set of four sensors. In the case the system is deployed in buildings with both large and small rooms, adding other positioning technologies to the system could be interesting, but this has not been considered yet.

On the other hand, the use of UWB-based positioning allows for the robust operation of the system, because with this technology there is no need for direct line of sight between tags and sensors, making it able to deal with objects blocking the line of sight. Additionally, to increase robustness the system includes a detailed spatial database of the building which can be derived from the building’s blueprint with the assistance of a configuration tool. Thanks to the details of this database, which include the location of walls, obstacles, points of interest, *etc*., and the use of the A* pathfinding algorithm, the system helps the user to avoid known obstacles more easily in comparison with other systems which try to detect objects in real-time, which could fail in the detection or force numerous route recalculations. More importantly, the SUGAR system is able to obtain shorter routes which avoid obstacles with enough anticipation and to keep a safe distance from them.

In addition to the features described in the previous paragraphs, several requirements are described in this paragraph that must be fulfilled in order to ensure the proper operation of the system, namely real-time operation, reusability, and providing a natural interface to the user. Firstly, the system must support real-time constraints; that is, not only does the visually impaired user have to receive frequent guidance updates but, more importantly, if the system detects any potential danger, it must be able to react in a timely manner. Although this is not described in great detail in the paper, all modules are configured with appropriate operation intervals which ensure the rapid response of the system. These intervals control how often position is estimated and instructions given to the user; therefore, their values have been set according to the typical travel speeds of users. Regarding reusability, the selected components of the system can be used in different scenarios in spite of the layout differences between buildings. After the initialization phase described later, where sensors and reference axes are calibrated and the spatial database is created, the accuracy provided by the system is quite satisfactory. As for the interface with the user, acoustic signals are employed. As will be explained later, in the SUGAR system a combination of voice commands and beep sounds is used.

**Figure 1:**
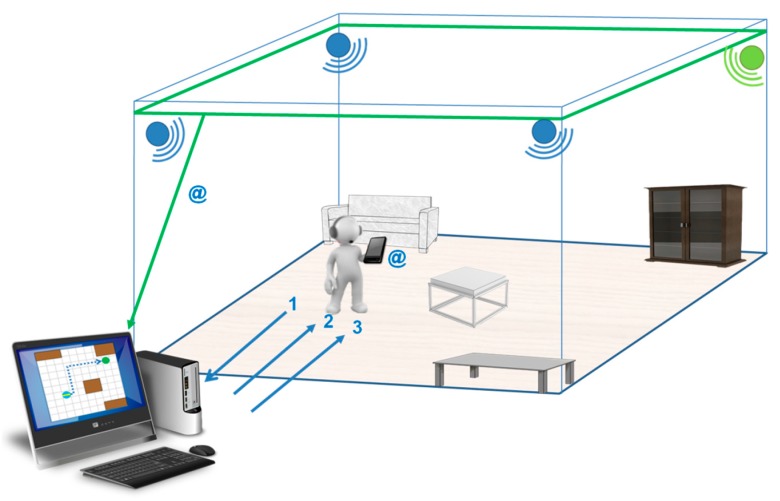
Overall description of SUGAR.

The overall operation of the system is illustrated in Figure 1. It describes a situation where a visually impaired user equipped with a smartphone, headphones, and a Ubisense UWB tag is in an unknown environment where the SUGAR system has been deployed. If they want to go from their current location to a specific place, they will have to select the location to visit from a list of points of interest played through the headphones (1). At this point, the system will obtain the path which the user has to follow (2). Along the way, their location and orientation is continuously tracked by wall‑mounted Ubisense UWB-based sensors and compass information from the smartphone. Simultaneously, they will be guided by different kinds of acoustic signals (3), which will help them to follow the calculated path and avoid obstacles during the journey.

**Figure 2:**
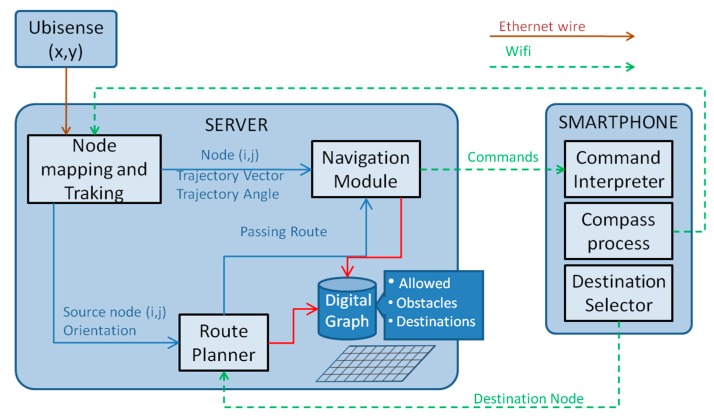
Architecture of the system.

Figure 1 also shows the physical components of the system. In addition to the aforementioned smartphone, UWB sensors, and the UWB tag embedded in the headphones, there is also a server which hosts several modules, as shown in Figure 2, that are in charge of the different tasks of the system. Most of the processing relies on this server, whereas the smartphone carried by the user has little computational load and is mostly used for user interaction tasks through the headphones. Communication between the different components, in turn, is conducted by using a wired Ethernet network (in the case of the UWB sensors) and a Wi-Fi network (for the communication between the server and the smartphone).

The architecture of the system is depicted in Figure 2, which also provides a good basis for explaining the system workflow. Assuming that the system has a digital map of the scenario in the form of a graph, where nodes in a grid are marked either as “walkable” (allowed nodes) or as obstacles, and that the user has already specified the destination, the sequence of activities which takes place is as follows:

- Ubisense UWB sensors continuously track the tag carried by the user; as a result, a sequence of locations in Cartesian coordinates is returned. Similarly, the orientation of the user is periodically measured by the smartphone.
- From the previous sets of measurements, the user’s location is then mapped to a node of the graph and their current direction of movement inferred. This is performed by the node mapping and tracking module.
- Once the source and destination locations (and orientations) are known, the route planner module selects the best route between them.
- As the user moves, the navigation module compares their location and trajectory with the previously calculated route and determines the actions that need to be taken in order to make sure that the user does not diverge from the target path.
- The smartphone receives the navigation commands via Wi-Fi connection and plays them through the headphones.

In the next subsections, the data model implemented, the initialization process, and each one of the modules and the technologies applied will be discussed.

### 3.1. Data Model

The system has a spatial database of the environment. This is the so-called *detail graph* where the environment is divided into rectangular or square nodes, which are marked either as walkable or as obstacles. The side lengths of the nodes are parameters and have been set approximately to a person's average stride length. This graph is essential for the proper operation of both the path planner and the navigation modules. However, there are other modules that provide or calculate data represented in other reference systems. The reference systems used are the following:

- Cartesian (x, y). It represents the x- and y-coordinates, in centimeters, of a user from the location set as the origin. Ubisense provides locations using the Cartesian coordinate system.
- Orientation. It represents the orientation of a user with north as the reference.
- Graph (*i, j*). Coordinates *i* and *j* determine which node of the *detail graph* the user is in.
- Symbolic. Certain areas of the environment can be assigned human-readable names. With this information, the system is able to inform the user about the room in which they are located or about the presence of dangerous areas such as stairs or transition areas such as doors or lifts.

### 3.2. System Installation and Initialization

This work uses the UWB-based Ubisense indoor positioning solution and, consequently, most of the system installation is related to the deployment of Ubisense components. For proper installation, the area must be divided into rectangular cells, which could be, for example, the different rooms of a building. Each cell requires the deployment of at least two sensors, with a typical configuration of four sensors, one at each corner of the cell. Sensors must be connected to an Ethernet switch, which is responsible for the communications with the system server where the Ubisense process is hosted. Further, additional direct wirings must be made between the sensors themselves. In this regard, one sensor is designated as the master sensor and all other sensors as slaves, requiring the connection of a timing cable from the master to each of the slaves. These cables are used by slave nodes to report the detection of tags to the master node, which will compute their location using the information received. Alternatively, if the master runs out of timing ports or if the cell is too large, some of the slaves can be connected to the cell’s master through intermediate sensors in a so-called daisy chain scheme. Ubisense’s system manuals, however, recommend keeping the number of hops between master and slaves as small as the physical constraints of the environment allow (direct cables can be used for distances up to 100 m). The other component of Ubisense technology, the tag, is embedded into the headphones that the visually impaired user will wear. Once all Ubisense components are deployed they must be configured using the software and following the instructions provided by the Ubisense Corporation.

The next installation step is to deploy a Wi-Fi network in order to allow the interaction between the smartphone carried by the user and the system server. This completes the physical deployment of the system components. In Section 4, an example of the deployment in a real scenario will be described. Once the system installation is completed, it must be initialized prior to its use. The initialization is accomplished by calibrating the aforementioned reference systems from a floor plan and creating the detail graph, which is explained in the following paragraphs.

The first step in the calibration process involves loading an image file with the floor plan of the building where the system has been deployed. With the digital map obtained, the administrator must calibrate the scale of the map so that it is possible to establish a relationship between the pixels of the map and the real distances of the environment. To this end, it is necessary to select two points on the map and enter the actual distance between them. Next, the origin must be set by selecting a point on the map which will be considered as the Cartesian coordinate (0, 0). Finally, the orientation reference system must also be calibrated by specifying north on the map. This step enables orientation readings to be transformed from the compass of the smartphone, which uses another different direction as the reference.

As for the creation of the graph, once the steps above have been performed, the system will display a grid in which the administrator will enter information about which areas are walkable and where the obstacles, the boundaries of the building, and the points of interest are delimited. The administrator must be cautious when placing obstacles, as their actual size may not correspond to that chosen in the grid due to the limited resolution of the grid. For safety reasons, the size selected for the obstacles must be greater than their real size in order to avoid accidents even when users are travelling by allowed nodes.

After this initialization phase, the *detail graph* can be obtained from the data entered. In addition, the reference systems are ready to deal with locations provided by Ubisense and orientation measured by the compass sensor integrated in the smartphone.

### 3.3. Location and Orientation Acquisition

UWB technology yields very good results for indoor positioning [9]. The main advantage of UWB for positioning is the short duration of UWB signals, which enables not only a high time resolution for pulse timing but also the possibility of distinguishing the direct signal from the signal reflections that usually occur in indoor environments. Several techniques are available to infer the position of a UWB emitting device. For the purpose of this work, Time Difference of Arrival (TDOA) and Angle of Arrival (AOA) techniques will be the most relevant. Using TDOA, position can be obtained by comparing pulse arrival times at different sensor devices with their respective antennas. Meanwhile, the AOA technique estimates the angle of the target with respect to each sensor device, provided they are equipped with an array of antennas.

In this work, the UWB-based Ubisense commercial solution is employed. According to the manufacturer, it can offer a precision of up to 15 cm with a 95% confidence interval in a three‑dimensional space by combining the aforementioned TDOA and AOA techniques. Since in real world deployments such high precision levels are hard to achieve, in the present work it was decided that the z-coordinate would be fixed and Ubisense would be allowed to focus on obtaining just the x‑ and y‑coordinates, thus improving precision. This is possible since the tag, weighing only 25 g, can be attached to the headphones used to give sound and voice commands to the user, and therefore its height above the floor has very little variation when the user is walking.

Regarding the acquisition of orientation data, the system runs a background process in the smartphone. This process measures the orientation of the user relative to north on the smartphone and delivers it to the system server. In the server, the value received has to be translated to the orientation reference system used in the digital map of the environment, where north does not exactly correspond.

A couple of problems were observed about the orientation acquisition from the compass of the smartphone. First, it was noticed that the compass returned bursts of erroneous data, typically one or two consecutive incorrect readings. Second, the compass was greatly affected by the near presence of large metallic objects. The first problem was easy to solve by discarding erroneous readings. This approach is feasible since the node mapping and tracking module, explained in the next section, computes location as the average of several measurements obtained during an interval, and consequently some of them may be discarded. Each reading from the compass sensor is compared with the previous valid reading; if the difference between them is greater than a certain threshold, it will be discarded provided that the maximum number of discards allowed for a single interval is not exceeded.

The problem with metallic objects, which cause magnetic field perturbations, is more complicated to deal with. Several possible solutions were proposed by Ubisense’s technical department. One of them consisted in using a tag with a directional antenna, which would have allowed orientation to be estimated with low precision but without large inaccuracies. This option was not chosen because it would have entailed an important effort, as Ubisense does not provide software or APIs for its implementation. A second option was to use two tags separated by a sufficient distance and to compute orientation according to the estimated position of each tag. In the case of a person, tags would have to be placed on each shoulder. This option was dismissed because previously performed precision tests comparing the location accuracy obtained by placing tags on different parts of the body showed that placing a single tag on the head (in headphones, cap, *etc*.) had a much better precision than placing it on a shoulder, an arm or the abdomen (because of the signal blocking caused by the human body). Although orientation precision would have been better, location precision, which is more important for the proper operation of the system, would have worsened.

The solution that was finally implemented is to estimate, whenever it is possible, orientation as a function of the trajectory of the user. This task is performed by the node mapping and tracking module described in the next section. 

### 3.4. Node Mapping and Tracking

Once location and orientation data are available, this information has to be processed before being used by the modules in charge of planning routes and guiding the user. Ubisense’s locations must be mapped to nodes of the *detail graph* and orientation must be estimated either from the readings of the compass or from the trajectory of the user. This is done by the node mapping and tracking module, providing data such as the current node where the user is located, a series of previously visited nodes, the orientation of the user, whether the user is moving or static, and, in the case they are in motion, the direction of the movement.

**Figure 3:**
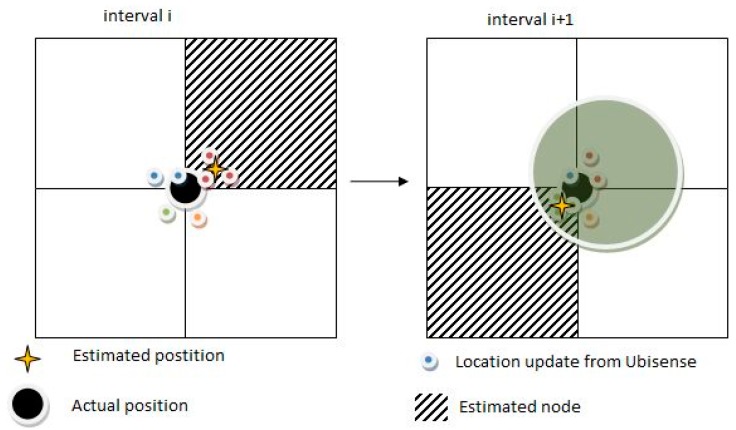
Movement detection. User is standing over the boundary between nodes, no movement is reported.

As for the selection of the current node, the node mapping module must accurately determine in which node of the *detail graph* the user is located. For this purpose, the module establishes a periodic interval during which it collects location updates from Ubisense and, after every interval, the node estimation takes place. The duration of this interval is parameterized and has been chosen to be approximately similar to an average stride time so that from one estimation to the next there is enough time for a person to move to another node. The calculation of the node where the user is located is conducted by checking the coherence of the movement with regard to the time window where samples are taken and calculating an average. The node is estimated with high precision, although in some cases where the user is located close to the boundary between two or more nodes it is possible that the node is assigned to any of the adjacent nodes. In any case, this is acceptable, since in this particular case the distance from the actual position to any of the nodes is quite short, and does not jeopardize the overall performance of the system. However, determining if a person is moving or not can be challenging because of this. If the user is standing close to a node boundary, it is possible that every time that a new mapping estimation is done the user is assigned to a different node and thus a false motion estimation is inferred. Similarly, if a user placed at one of the sides of the node moves to another of the sides, it is likely that the estimated node remains the same, which is right, but no movement is reported. In order to solve these problems several movement estimation methods were studied. Finally, it was decided to ignore the node mappings and instead use the average position estimated during consecutive intervals. As can be seen in Figure 3, a circle of a certain radius, centered on the previous location estimated, is drawn; if the new estimation is contained within this circle the user will be considered to have stopped, and otherwise to be moving. In this figure, during interval *i* the user is standing on the boundary between four nodes. From a location point of view, it is not very important to which of the adjacent nodes it is assigned by the mapping module, as the user is equally distant to all of them. However, if the user is assigned to a different node after interval *i + 1*, the mapping module will not report that the user is moving since it can observe that the estimated locations at intervals *i* and *i+1* are quite close within the maximum distance represented by the circle in the right part of the figure.

Finally, the mapping module provides orientation information in the form of the trajectory vector and the trajectory angle. The trajectory vector is a vector pointing from the center of the previous node to the center of the current node, therefore, it represents the current movement direction. The trajectory angle, in turn, measures the orientation of the user relative to north of the digital map of the environment. There are two types of trajectory angles depending on the source used to calculate them. The first is the Vector Trajectory Angle (VTA), which is calculated from the trajectory vector, and the second is the Sensor Trajectory Angle (STA), obtained from the processed sensor (compass) readings of the smartphone. Given the typical inaccuracies from the compass readings, the preferred angle is the VTA; however, this angle is only valid when the user is moving. Consequently, when the user moves and the trajectory vector is valid, the VTA is used to measure orientation and, when the user is stopped, the STA must be used.

It must be noted that calculating the user orientation in this way results in a slight loss of precision. However, this fact will not hamper the usability of the system. In a worst case scenario, since the guidance relies more heavily on location than on orientation, if a user deviates slightly from the computed path, as soon as they are mapped to an adjacent node to the target node, the trajectory will be corrected.

### 3.5. Route Planning

**Figure 4:**
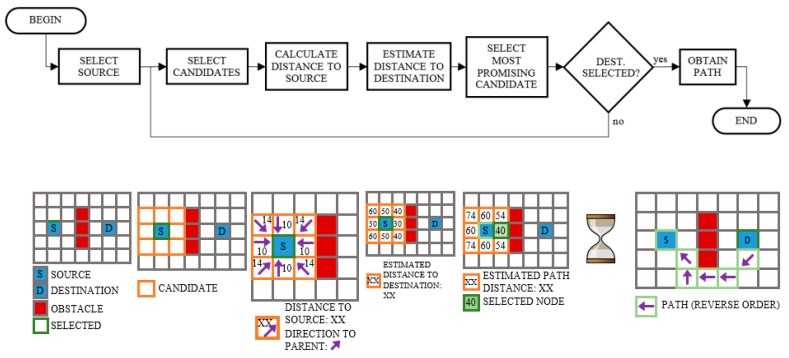
A* workflow.

Given both the desired target location and the current location, the route planner is able to obtain the route between them. For this purpose, the A* algorithm [26,27] is applied, which is a very efficient graph search algorithm widely used in pathfinding. As depicted in Figure 4, the algorithm iteratively selects one node that has the shortest estimated distance to the destination, which is the source or current node, as the first selected node, and then each reachable neighbor node from the selected one becomes a candidate to be the next selected node (if it has not been selected before). For each candidate node, the distance to the origin node via the selected node is calculated and this node becomes the parent (previous hop in the path) of the candidate if it provides a new path between both nodes or a better path than that obtained in previous iterations. In addition, the distance of the candidates to the destination node is estimated using a heuristic (explained in the next paragraph). From all of the candidates the best one is selected (whose sum of distances to the source and destination nodes is smaller, that is, the node which would offer a shorter path if it was selected) and the process is repeated until the destination node is reached.

The main difference of the A* algorithm to other graph search algorithms such as Dijkstra is the use of a heuristic to estimate the distance from the candidate nodes to the destination node, which has the advantage of first exploring more likely paths to the destination and thus saving time with paths that are not promising. The heuristic employed by the path planner is the Manhattan method, which estimates the distance to the destination as the number of nodes that has to be traversed using only horizontal or vertical movements. Although the use of the Manhattan method will lead in rare occasions to the shortest path not being found, the resulting path will be almost as short as the optimal and the computation time will be minimal.

In order to obtain the path between the current node and the destination node, the parent nodes, which indicate the previous hops in the path, are followed in reverse order, that is, starting from the destination node. However, this path is not directly passed as a parameter to other modules but rather it is previously processed. The output given to the navigation module is the passing route, which consists of reference points, a list of allowed nodes and a list of boundary nodes. The reference points divide the path into straight segments and when a user reaches any of them they will be asked to change direction. In addition, since the calculated path is too narrow, at just one node in width, more nodes will be added to the list of allowed nodes according to two configurable parameters which specify the desired path width and the minimum allowable distance to obstacles.

Finally, it should be noted that the passing route will not only be calculated once at the start of the itinerary. It will also be re-calculated if the user visits a node which is not in the list of allowed nodes of if the user deviates from the established direction by a large amount, as mentioned in the next subsection.

### 3.6. Navigation

The navigation module is in charge of ensuring that the user follows the calculated path safely, staying always within the list of allowed nodes in the passing route. To this end, the module will issue commands to the user which will guide them from one reference point to the next, where new directions will be given.

**Figure 5:**
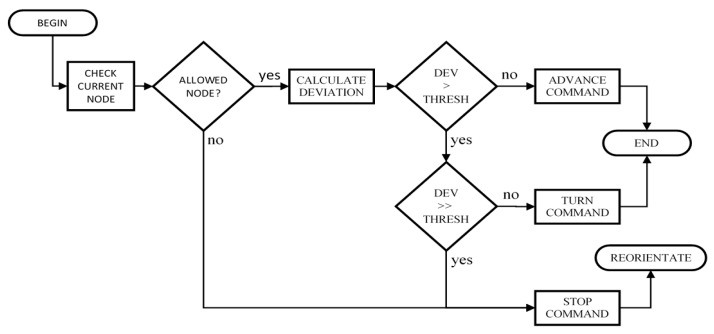
Guidance process to the next point of reference.

The operation of the module to get the user to the next reference point is depicted in the flowchart of Figure 5. Firstly, the module checks the user’s position and, should they be in a not allowed node (obstacle node), a stop command will be issued and afterwards the user will be reoriented toward the next reference point. This reorientation implies that the route planner recalculates the route to the destination, since there are no guarantees that the path to the next reference point is free of obstacles. In the case the user is in an allowed node, then their trajectory (trajectory vector) is compared with the objective trajectory to the next reference node. If the deviation is smaller than a certain threshold, an advance command will be issued. On the contrary, if the deviation is bigger than the threshold, an order asking them to turn is sent. There is also the possibility that the deviation from the path is too large. In this case, the procedure would be similar to that explained above for nodes not allowed: the user would be asked to stop and they would be reoriented.

### 3.7. Human–Machine Interaction

The commands sent from the server via Wi-Fi are received by the smartphone, where they are played through the headphones. Two different types of commands can be received, namely verbal commands (for selecting the destination, starting the journey, and to stop once final destination is reached) and soft beeps for guidance. In order to determine how to make use of them, an association for the blind, *Asociación Retina Navarra* [28], jointly collaborated with us for a usability study. According to their feedback, during guidance there was a preference for soft beeps over speech commands. This is in line with the results presented in other previous research, which argued that interpreting speech sounds adds more cognitive load than non-speech sounds [23]. In the SUGAR system, additional verbal commands will be used occasionally to inform the user about events of major relevance such as the stop command to avoid a collision, the presence of stairs, or any other potential danger nearby, as well as arrival to a destination point. In order to provide the system with more extensibility, the commands are not preloaded on the smartphone, but rather it is just an interpreter of the text messages received. This allows as many commands to be added as desired without having to update the application loaded on each smartphone being used.

As for the beep sounds, they are used to indicate the direction that should be followed depending on the sequence of beeps produced. Two types of beeps with different acoustic frequencies are used: ticks and tocks. When they alternate evenly, the user is asked to move forward. If the user has to modify their direction, only ticks or tocks will be played if the turn is to the left or to the right, respectively. In this case, the frequency of the repetition of the beeps determines how pronounced the turn is. That is, if the time separation between ticks (or tocks) is long the user will keep advancing, slightly modifying their trajectory to the requested direction. Conversely, if beeps are more frequent the user will perform a more pronounced change in direction. When moving forward the separation between beeps does not have any effect on the trajectory or speed of the user. In this case the rate of beeps is set in order to make the user feel comfortable, since a rate that is too high may be annoying, whereas, wearing headphones but hearing no sound instruction for long periods of time may make the user feel dubious and unsure.

Initially the interaction with the user was implemented by means of beeps reproduced in either ear, depending on the direction the user was requested to turn. The tick-tock beep sequence finally used gives, on the one hand, the possibility of using just one earpiece speaker instead of two and, therefore, not covering one the ears. This is important for blind or visually impaired people as they mainly rely on their auditory system to interact with other people or the environment. An interesting addition to the system would be the use of bone conducting headphones, which would allow both ears to be uncovered. On the other hand, another reason for using ticks and tocks was its ease of comprehension by users.

In [23], spatialized audio (sounds that appear to come from the direction of the next waypoint) was proven to be superior to speech for guidance. A small percentage of the participants in the tests performed worst on the first trial with spatialized audio, although they did well on subsequent trials. In the case of the SUGAR systems, aimed at events such as conferences, users will make sporadic use of the system, and it is important to use a beep sequence that can be easily interpreted with a minimum instruction time. This may be especially important when taking into account that users of the system may be elderly people, whose auditory or cognitive capabilities can be deteriorated. In addition, spatialized audio requires precise measurements from the compass. However, due to the measures taken in order to cope with the inaccuracies of the compass, the orientation precision is not sufficient enough to benefit from this approach.

To conclude the interaction with the user, the way in which the user selects the destination must be mentioned. In order to perform this task, the user has to first open the SUGAR application on the smartphone (in the prototype this was done by a long press on one of the side buttons of the phone). After this, the names of all potential destinations are read to the user and, in order to select one of them, they must double tap the screen after hearing the destination. From this point on, the user will be guided by the acoustic signals explained above.

## 4. Evaluation in a Real Scenario: Results and Discussion

The system presented in this paper was tested in a real scenario. A test was carried out in conjunction with the company Life Quality Technology (LQT) [29], which has added the SUGAR system to its product portfolio. The test consisted of, on the one hand, a verification of the proper operation of the system by means of its utilization by a blind person (untrained in the use of the system), and, on the other hand, obtaining some accuracy statistics. The verification of the system was successful, and there is a video showing a real demonstration of the operation of the system by a blind person travelling to several points of interest at the Asociación Accesibilidad Universal premises in Pamplona (Spain) [30]. The rest of the section is devoted to the accuracy tests that were performed, describing how the system was deployed, calibrated, and configured as well as discussing the obtained results. In addition, some further testing done at a later date is also described.

**Figure 6:**
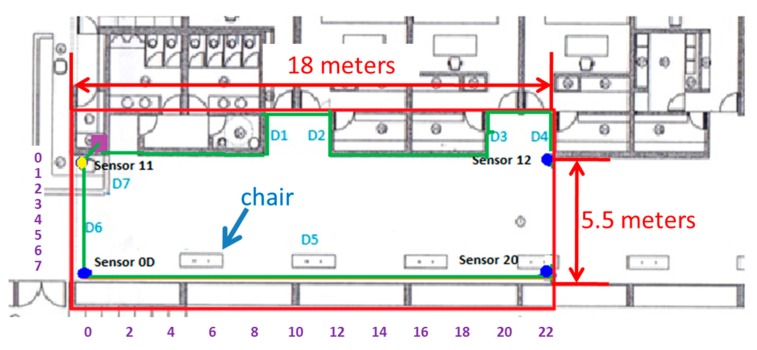
Map of the real scenario and the deployment of the devices.

Figure 6 illustrates the map of the testing area within the facilities where the system was deployed and tested. Over the map, dots and lines represent the different components of the system and how they are connected with each other. The Ubisense sensors are located in the corners of this area and are represented by four dots: the yellow dot represents the master sensor, whereas the blue dots denote the slave sensors. The magenta point is used to mark the position of several elements that can be placed together (the switch, the router, and the SUGAR–UWB–Android server). Finally, the network cables that connect the different components (the sensors and the server) to the switch are depicted as green lines.

As was established in Section 3, the system uses a spatial database characterizing the environment where the covered area is mapped into square nodes. Therefore, once the sensors are installed, the next step is to measure two reference points that will be matched with the corresponding points in the digital map in order to calibrate the scale. As a rule of thumb, and in order to simplify the testing procedure, the tiles of the floor (with a shape of 40.5 × 40.5 cm) were used as a pattern for the node’s size. That is, a node was considered as a square composed of four tiles, with a size of 80.1 × 80.1 cm. This consideration leads to a mapped area of 22 columns and seven rows (represented in the system as a 22 × 7 matrix and represented in Figure 6 with numbers in purple) corresponding to a covered area of approximately 18 × 5.5 m (also depicted in the figure).

In the testing process, the Ubisense tag (which is embedded into the headphones) was adjusted to the height of the person who carried the tag. In order to verify the accuracy and the effectiveness of the Ubisense system, some statistics, such as the mean of the error value and the standard deviation for these errors of the coordinates (x, y), were calculated as metrics of the system.

**Figure 7:**
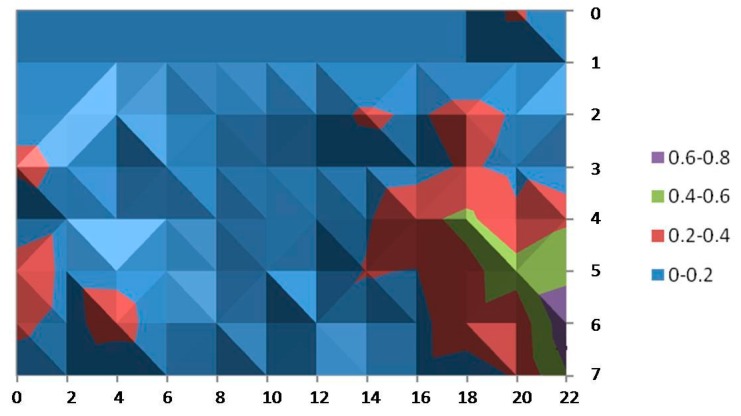
Average error for the x-coordinate.

Figure 7 shows the average error of x-coordinate over the covered area. In this figure, each point represents the average error of the x-position, that is, for each point, several measurements of the x position given by Ubisense were taken, and then the average error compared with the real physical position was calculated. The error values are interpolated in the figure and depicted in a contour chart with shading where color bands are separated by contour lines, clustering values in ranges of 0.2 m. The hill-shading technique is used to create the impression of relief in order to remark the highest values. Consequently, the different shades of a particular color correspond to the same error band.

**Figure 8:**
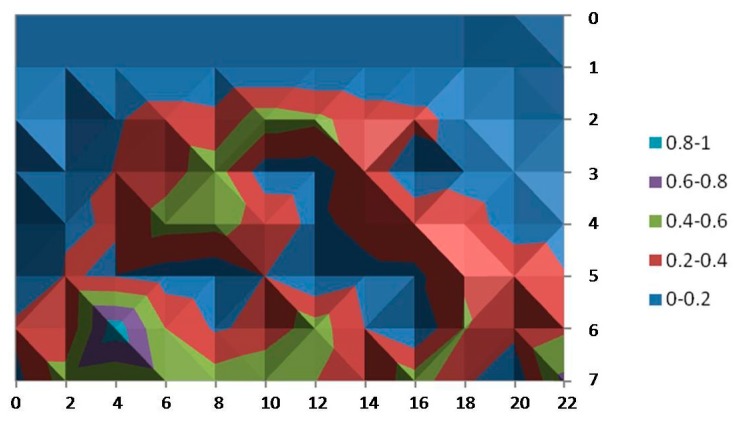
Average error for the y-coordinate.

In the same way, Figure 8 shows the average error of the y coordinate over the considered area. It is observed that, in this scenario, Ubisense seems to be more stable for the x-coordinate: In almost the entire area, the average error has values below 20 cm and just a small portion of the area is in the highest range of 60–80 cm. On the other hand, the average error calculated for the y-coordinate shows higher values (ranging up to 60 cm and even an isolated small area with values of 80 cm can be noticed). In addition, these higher values are not enclosed in a small area and can even be found in the central (and probably more walkable) area.

The reason for these higher errors in a few small areas and quite possibly for the difference between the axes is due to the dimensions of the room. The width of the room (y-axis) is only 5.5 m long; however, triangulation using the AOA technique requires a separation of at least 10 m between sensors in order to provide more accurate results. In spite of this fact, the accuracy is good enough in most of the area of the cell because the TDOA technique is also used for position estimation.

**Figure 9:**
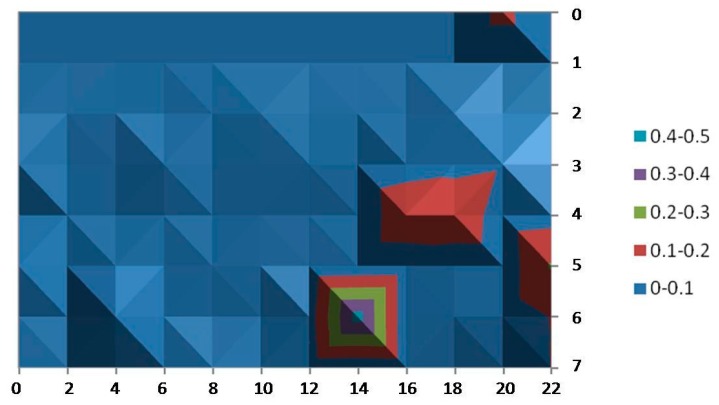
Standard deviation of the samples taken for the x-coordinate.

**Figure 10:**
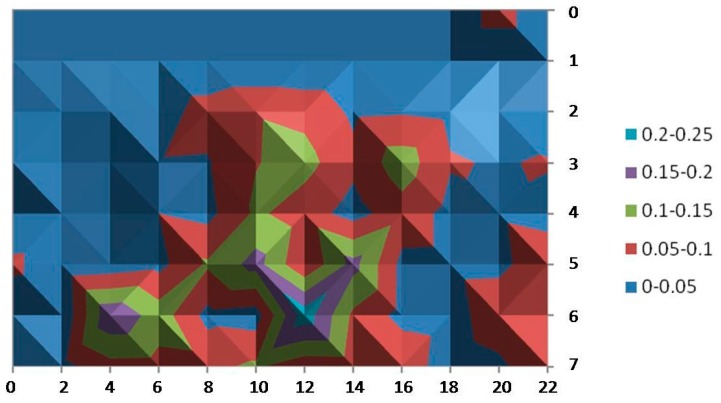
Standard deviation of the samples taken for the y-coordinate.

Figure 9 and Figure 10 provide the standard deviation for the x- and y-coordinates. The results reveal that the measurements of our tests vary little among almost all of the covered area: between less than 10 cm and 50 cm (just at isolated points) for the measures of the x-coordinate and even better for the y‑coordinates (all of them are below 0.25 cm).

After the realization of the test it was concluded that the areas with higher positioning errors could hamper the usability of the system. Marking those areas as obstacles was examined as a possible solution. Finally, another possibility was considered: the use of a second tag in order to improve accuracy.

**Figure 11:**
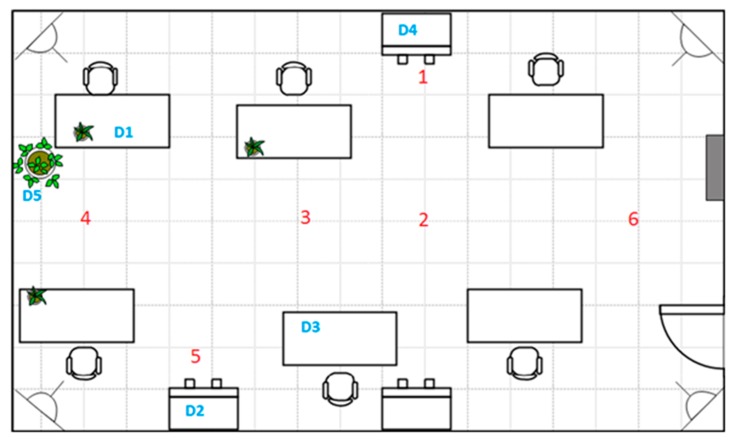
Office layout and points selected for measurements.

Another test was performed in the facilities of LQT comparing the accuracy performance using one and two tags. Six different points of an office, shown in Figure 11, were selected to evaluate the location error for both cases, considering different situations such as points close to metal objects (points 1 and 5), where the worst performance is expected, points in the middle of the room (points 2 and 3), and points at the ends of the room (points 4 and 6). Four samples were taken for each selected point (similar to the number of samples processed by the node mapping module during an estimation interval) and the average errors were calculated, as shown in Table 1.

**Table 1:** Average accuracy errors for one and two tags (in meters).

Point in Room	1 Tag—x-Axis	2 Tags—x-Axis	1 Tag—y-Axis	2 Tags—y-Axis
1	0.381	0.08175	0.4715	0.17225
2	0.1595	0.20725	0.2625	0.10125
3	0.09875	0.16225	0.09075	0.04875
4	0.11525	0.2825	0.17175	0.17525
5	0.2155	0.13125	0.072	0.08825
6	0.2115	0.1615	0.28775	0.08875

It can be observed that the highest errors obtained correspond to the one tag case and that the greater the error is the more it improves using two tags. This is especially noticeable in node 1 where the y‑coordinate error improves from 47.15 cm to 17.22 cm in the y-axis and from 38.1 cm to just 8 cm in the x-axis. On the contrary, those points which had an error below 20 cm showed little or no improvement with the use of two tags.

From the tests explained in this section it can be concluded that the SUGAR system offers quite high accuracy. It requires the use of two tags in order to avoid high error values in some areas of the scenario which, nevertheless, are small in size. In addition, the accuracy of the system improves for larger areas (that are not too narrow in one of their dimensions), which is a major advantage in comparison with other technologies based on the placement of a large number of devices such as RFID or NFC.

## 5. Conclusions

In this paper, SUGAR, an indoor navigation system for visually impaired people, is presented. The architecture of the system has been described as well as different tests in a real-world scenario. The system is in charge, on the one hand, of locating the user and, on the other hand, of guiding them to a selected destination (or through a route with several points of interest). The location of the user is performed with a high level of accuracy due to the use of UWB positioning techniques in a two‑dimensional space. As for the navigation process, a digitalization of the floor plan of a building is done through a process that results in the creation of a spatial database that contains information about obstacles, points of interest, and walkable areas. The A* algorithm is applied to the information of the database in order to obtain the appropriate path to the destination. After further processing of the path, a passing route with additional guidance information is obtained. Finally, the system provides all necessary information to the user by means of voice commands and acoustic signals to ensure that the user is properly guided to the destination.

This proposal is a step forward in guidance for the visually impaired, as it manages to combine accuracy, robustness, and ease of use in a system with a simple installation. It has been designed to be installed in large public buildings, although it can be deployed in a great variety of scenarios. In addition, the system has been included in the product portfolio of an enterprise dedicated to improving the lives of elderly people by means of new technologies (LQT), which will be in charge of performing intensive formal validation tests of the system.
